# Relationship between Random Blood Glucose, Fasting Blood Glucose, and Gensini Score in Patients with Acute Myocardial Infarction

**DOI:** 10.1155/2019/9707513

**Published:** 2019-10-15

**Authors:** Yuhan Qin, Gaoliang Yan, Yong Qiao, Changle Ma, Juchuan Liu, Chengchun Tang

**Affiliations:** ^1^Medical School of Southeast University, Nanjing 210009, China; ^2^Department of Cardiology, Zhongda Hospital Affiliated to Southeast University, Nanjing 210009, China

## Abstract

**Objective:**

To examine the relationship between admission random blood glucose (RBG), fasting blood glucose (FBG), and Gensini score in patients with acute myocardial infarction (AMI) to clarify the effects of RBG and FBG on the severity of coronary artery disease.

**Method:**

A total of 958 consecutive AMI patients who underwent emergency coronary angiography at the Cardiology Department of Zhongda Hospital (affiliated with Southeast University) were enrolled in this study from January 1, 2016, to December 31, 2018. The Gensini score of each patient was calculated according to the results of coronary angiography. The RBG, FBG, baseline data, hematological indexes, echocardiography parameters, coronary angiography data, and the use of intra-aortic balloon pump (IABP) were recorded. Patients with an RBG level >11.1 mmol/L were classified into the stress hyperglycemia group, and those with an FBG level >7.0 mmol/L were classified into the elevated FBG group. The Gensini scores in the stress hyperglycemia and elevated FBG groups were compared to those in the control group, and correlations between the RBG and FBG levels and the Gensini scores of AMI patients were evaluated. Independent risk factors for the Gensini score were analyzed by multiple linear and multiple logistic regression analyses.

**Results:**

The Gensini scores of the stress hyperglycemia group and the elevated FBG group were higher than those of the control group. RBG and FBG were positively correlated with the Gensini score, and there were significant differences between RBG and FBG in different Gensini score groups. After adjusting for confounding factors, multiple linear regression analysis showed that sex, diabetes, estimated glomerular filtration rate (eGFR), and FBG were independent risk factors for the Gensini score. Multiple logistic regression analysis showed that age and FBG were independent risk factors in group 2 compared to group 1, eGFR and FBG were independent risk factors in group 3, and eGFR and FBG were independent risk factors in group 4. Diabetes and RBG were not independent risk factors for the Gensini score.

**Conclusion:**

The Gensini scores of patients in the stress hyperglycemia group and the elevated FBG group were significantly higher than those in the control group. RBG and FBG were positively correlated with the Gensini score in AMI patients, and FBG was an independent risk factor for the Gensini score in AMI patients.

## 1. Introduction

Diabetes is an independent risk factor for coronary artery disease (CAD), and CAD is the main cause of death in diabetes patients. Compared to the nondiabetic population, diabetes is associated with a 2-3-fold increase in the risk of cardiovascular disease and mortality due to cardiovascular disease [1]. Diabetics without a history of myocardial infarction and nondiabetic patients with a history of myocardial infarction have approximately the same risks of acute myocardial infarction (AMI) and death from CAD [2]. Patients with diabetes tend to have more extensive, diffuse, calcified, and severe coronary artery lesions [3] and a higher Gensini score than nondiabetics [4]. AMI patients also have a high prevalence of diabetes, and the INTERHEART study, performed in 52 countries, indicated that diabetes contributed 10% of the attributable risk in newly diagnosed AMI patients [5]. The DECODE study indicated that abnormal glucose tolerance also increased the risk of cardiovascular disease and led to a poor prognosis [6]. Patients with an impaired fasting glucose (IFG) level and impaired glucose tolerance (IGT) have more severe coronary artery lesions [7]. Hyperglycemia is also an independent risk factor for both in-hospital and long-term poor prognosis in AMI patients [8]. Hyperglycemia is related to diabetic microvascular complications, including the incidence of diabetic retinopathy and diabetic nephropathy, the latter of which can be reduced by treating hyperglycemia [9]. This study examined the relationships between admission random blood glucose (RBG), fasting blood glucose (FBG), and Gensini score and evaluated the effects of RBG and FBG on the severity of CAD in AMI patients.

## 2. Materials and Methods

### 2.1. Study Population

A total of 958 consecutive AMI patients who underwent emergency coronary angiography at the Cardiology Department of Zhongda Hospital (affiliated with Southeast University), from January 1, 2016, to December 31, 2018, were enrolled in this study. The study population had an average age of 62.51 ± 13.49, and 744 (77.7%) were male. A total of 265 patients (27.7%) suffered stress hyperglycemia, and 589 (61.5%) had an elevated FBG level. Patients with AMI who underwent emergency coronary angiography and were aged 18–80 years were enrolled. The exclusion criteria were as follows: an allergy to iodine or iodine contrast agent, severe hemodynamic instability, severe liver and kidney dysfunction, severe infectious diseases, malignant tumors, severe blood system diseases, and incomplete data. All patients provided written informed consent before enrollment in the study.

### 2.2. Research Method

Baseline data, including sex, age, height, weight, systolic blood pressure (SBP), diastolic blood pressure (DBP), and Killip class, as well as previous and personal histories, including hypertension, diabetes, previous myocardial infarction (MI), cerebral infarction, chronic kidney dysfunction (CKD), and smoking history, were recorded. Hematological examination indexes, including RBG, FBG, troponin I (TNI), creatine kinase-muscle/brain (CK-MB), myoglobin (Myo), B-type natriuretic peptide (BNP), routine blood tests, liver and kidney function, serum lipids, echocardiography parameters, medications, coronary angiography data, and the use of an intra-aortic balloon pump (IABP) or coronary artery bypass grafting (CABG), were recorded.

#### 2.2.1. Coronary Angiography

Left and right coronary angiographic examinations were performed via radial artery puncture; multiposition projection was performed according to Judkins' method. The records were diagnosed independently by at least two interventional physicians blinded to the other data of the patients; a third physician performed the analysis when necessary.

#### 2.2.2. Stress Hyperglycemia

The American Diabetes Association (ADA) defines stress hyperglycemia as an FBG level >6.9 mmol/L or an RBG level >11.1 mmol/L with no history of diabetes [10]. In this study, we defined an RBG >11.1 mmol/L as stress hyperglycemia, regardless of the history of diabetes [10]. RBG was tested once patients entered hospital, and FBG was tested in the morning of the next day.

#### 2.2.3. Gensini Score

The degree of stenosis and the coronary artery lesion site were scored as follows. The degree of stenosis score was multiplied by the lesion site score, and the sum of the lesion score was taken as the final Gensini score [11] (Table 1). The Gensini score was defined by two separate doctors.

### 2.3. Statistical Analysis

SPSS 19.0 and MedCalc v18.11.3 software were used for all statistical analyses. Count data are expressed as cases and percentages, and the *χ*^2^ test was used for analysis. Numerical data are expressed as mean ± SD and were compared using the independent sample *t* test. Nonnormally distributed numerical data are expressed as the median and 25th–75th interquartile range and were compared using a rank-sum test. Spearman correlation analysis was used to evaluate the correlations between the RBG, FBG, and Gensini score. Multiple linear regression analysis and logistic regression analysis were performed to examine independent risk factors for Gensini score as continuous variables and hierarchical variables, respectively. In all analyses, *P* < 0.05 was taken to indicate statistical significance.

## 3. Results

### 3.1. Comparison of the Baseline Data, Hematological Indexes, Coronary Angiography Data, and Gensini Score in the Hyperglycemia Group and the Nonhyperglycemia Group

There were significant differences in sex, age, hypertension, diabetes, previous MI, cerebral infarction, CKD, smoking, Killip class I, Killip class IV, BNP, white blood cell (WBC) count, alanine aminotransferase (ALT), RBG, estimated glomerular filtration rate (eGFR), FBG, triglycerides (TG), total cholesterol (TC), high-density lipoprotein-cholesterol (HDL-C), low-density lipoprotein-cholesterol (LDL-C), left ventricular ejection fraction (LVEF), clopidogrel, ticagrelor, angiotensin-converting enzyme inhibitors (ACEI)/angiotensin II receptor blockers (ARBs), IABP, single-vessel lesion, double-vessel lesions, left main lesion, and Gensini score between the hyperglycemia group and the nonhyperglycemia group (*P* < 0.05). There were no significant differences in height, weight, body mass index (BMI), SBP, DBP, Killip class II, Killip class III, TNI, Myo, CK-MB, hemoglobin (Hb), platelets (PLT), aspartate aminotransferase (AST), serum creatinine (Scr), uric acid (UA), aspirin, *β*-blocker, statin, calcium channel blocker (CCB), furosemide, antisterone, CABG, triple-vessel lesions, and number of stents between the hyperglycemia group and the nonhyperglycemia group (*P* > 0.05) (Table 2).

### 3.2. Comparison of Baseline Data, Hematological Parameters, Coronary Angiography Data, and Gensini Score in the FBG Elevated Group and the Non-FBG Elevated Group

There were significant differences in sex, BMI, diabetes, cerebral infarction, smoking, Killip class I, Killip class IV, TNI, Myo, BNP, WBC, RBG, ALT, AST, FBG, HDL-C, LVEF, clopidogrel, ticagrelor, ACEI/ARB, furosemide, antisterone, IABP, single-vessel disease, triple-vessel disease, number of stents, left main lesion, and Gensini score between the elevated FBG group and the non-FBG elevated group (*P* < 0.05). There were no significant differences in age, height, weight, SBP, DBP, hypertension, previous MI, CKD, Killip class II, Killip class III, CK-MB, Hb, PLT, Scr, eGFR, UA, TG, TC, LDL-C, aspirin, *β*-blocker, statin, CCB, CABG, or double-vessel disease (*P* > 0.05) (Table 3).

### 3.3. Correlation Analysis of RBG and FBG with Gensini Score in AMI Patients

Spearman correlation analysis showed a positive correlation between RBG and Gensini score (*r* = 0.182, *P* < 0.001). In addition, there was a positive correlation between FBG and Gensini score (*r* = 0.171, *P* < 0.001).

According to the quartile method, we divided the patients' scores into four groups based on the Gensini score: group 1, Gensini score ≤ 37; group 2, 37 < Gensini score ≤ 60; group 3, 60 < Gensini score ≤ 88; and group 4, Gensini score > 88. The correlation coefficient between the RBG and the Gensini score was highest in group 1 (*r* = 0.298, *P* < 0.001).

### 3.4. Mean and Variance Analysis of RBG and FBG

**Figure 1:**
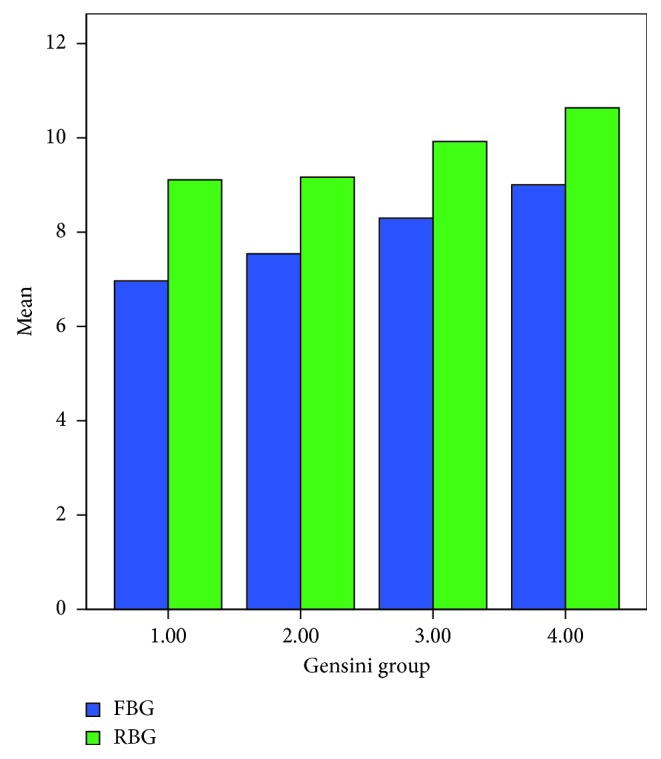
RBG and FBG mean distributions in different Gensini score groups.

Both RBG and FBG increased with the increasing Gensini score. The mean values of the RBG in groups 1–4 were 9.08, 9.25, 10.19, and 10.83 mmol/L, respectively. In addition, the mean values of the FBG were 7.29, 7.64, 8.20, and 9.01 mmol/L, respectively (Figure 1).

A one-way analysis of variance showed significant differences in RBG and FBG between the different Gensini score groups. The least significant difference (LSD) test showed that the RBG was significantly higher in groups 3 and 4 than in group 1 (*P*=0.020 and *P* < 0.001, respectively). Furthermore, the FBG was significantly higher in groups 3 and 4 than in group 1 (*P*=0.020 and *P* < 0.001, respectively). The FBG was higher in group 4 than in groups 2 and 3 (*P*=0.001 and *P*=0.04) (Table 4).

### 3.5. Multivariate Linear Regression Analysis

After adjusting for age, BMI, hypertension, CKD, smoking history, and serum lipid level, multiple linear regression analysis of the Gensini score as a continuous variable showed that sex, diabetes, eGFR, and FBG were independent risk factors related to the Gensini score (*r* = 9.770, 8.366, −0.165, and 1.540, respectively; and *P*=0.015,  0.043,  0.044,  and 0.039, respectively). RBG was not an independent risk factor related to the Gensini score (*r* = 1.145 and *P*=0.112) (Table 5).

Multiple logistic regression analysis showed that age and FBG were independent risk factors for the 37 < Gensini score ≤ 60 group (*P*=0.001 and *P*=0.037, respectively) compared to the Gensini score ≤ 37 group. The eGFR and FBG were independent risk factors for the 60 < Gensini score ≤ 88 group (*P*=0.014 and *P*=0.001, respectively). Furthermore, the eGFR and FBG were independent risk factors for the Gensini score > 88 group (*P*=0.005 and *P* < 0.001, respectively). Neither diabetes nor RBG was an independent risk factor for Gensini score (Table 6).

## 4. Discussion

This study explored the correlation between blood glucose and the severity of coronary lesions in AMI patients. Our research showed that 265 of 958 AMI patients had admission hyperglycemia, which accounted for 27.66% of all AMI patients. Besides, 589 patients had elevated FBG, nearly 1.6 times as many as patients with normal fasting blood glucose. Statistical analysis showed that FBG rather than RBG is an independent risk factor for the Gensini score. Multivariate linear regression analysis showed the regression coefficient for FBG to be 1.6.

The incidence rate of diabetes is increasing worldwide due to population aging, poor dietary patterns, and lack of exercise [12]. Diabetes mellitus is an important risk factor for cardiovascular disease, with diabetes mellitus patients showing a 2–4-fold higher risk of cardiovascular disease than people with normal blood glucose levels. More than 70% of diabetes patients over 65 die from cardiovascular and cerebrovascular diseases [13]. One-third of patients who undergo coronary intervention are diabetic, and a quarter of patients treated with CABG have a history of diabetes [14]. Coronary lesions tend to have greater inflammatory cell infiltration and more diffuse lesions in diabetic patients [15]. Diabetes is a risk factor for AMI, and diabetic patients with AMI have more severe lesions and an increased risk of poor short-term and long-term prognoses [16].

Stress hyperglycemia refers to transient hyperglycemia that occurs during the course of disease. The ADA defines stress hyperglycemia as an FBG level >6.9 mmol/L or RBG level >11.1 mmol/L without diabetes [10]. Stress hyperglycemia is closely related to mortality in AMI patients. In a meta-analysis involving 1856 AMI patients [8], the relative risk of in-hospital death in nondiabetic patients with stress hyperglycemia was 3.9-fold higher than that in patients with normal blood glucose levels (95% CI = 2.9–5.4), and diabetic patients with stress hyperglycemia were 1.7-fold more likely to die in hospital than those with normal blood glucose levels (95% CI = 1.2–2.4). Approximately half of all patients with ST-segment elevation MI (STEMI) are admitted with stress hyperglycemia [17], which can increase the MI area, severity of CAD, and mortality, leading to other adverse outcomes [18, 19]; decreased left ventricular systolic function may also be found [20]. The IABP-SHOCK II study showed that, in AMI patients with cardiogenic shock, RBG was an independent predictor of 30-day and 1-year mortality after MI. The 30-day mortality rates of patients with an RBG level ≥11.5 mmol/L and an RBG level <11.5 mmol/L were 47.7% vs. 36.5%, respectively (*P* = 0.004), and the corresponding 1-year mortality rates were 57.7% vs. 47.1%, respectively (*P*=0.011) [21]. Among acute coronary syndrome patients, patients with stress hyperglycemia had a higher Gensini score, an increased incidence of 6-month major adverse cardiac events and an increased incidence of in-hospital arrhythmia [22]. RBG interferes with blood flow in the culprit vessel and is linearly correlated with the TIMI blood flow index [23]. Some studies have indicated that RBG on admission is closely related to mortality in nondiabetic patients [24].

AMI is a life-threatening acute disease, in the acute phase of the disease, the accumulation of oxidative stress, cytokines, catecholamines, cortisol, and inflammatory markers results in a decrease in gluconeogenesis and an acceleration of glycogen decomposition leading to hyperglycemia [25]. Hyperglycemia is also associated with endothelial barrier injury, activation of NF-kappa-b, impaired endothelium-dependent vascular diastolic function, and the overexpression of adhesion molecules such as intercellular adhesion molecules, vascular cell adhesion molecules, and e-selectin [26, 27]. Catecholamines, cortisol, and other hormones are also increased, leading to increased glucose utilization, which is conducive to cell hibernation and prevents further apoptosis in AMI patients [28]. Previous animal studies demonstrated that SGLT2 inhibition reduced the mortality of diabetic rats after MI through cardiac energy metabolism and the protective modification of antioxidant proteins, suggesting that hyperglycemia is related to a disturbance in energy metabolism and oxidative stress during AMI [29]. In patients with abnormal glucose tolerance and diabetes mellitus, catalase, superoxide dismutase, glutathione (GSH), GSH reductase, and GSH peroxidase were negatively correlated with the Gensini score, suggesting that oxidative stress is involved in the mechanism underlying the aggravation of CAD by hyperglycemia [30].

IFG refers to a fasting glucose level of 6.1–7.0 mmol/L. In a 5-year follow-up of newly diagnosed IFG patients at the Singapore National Hospital, 20 (0.9%) of the 2295 IFG patients developed AMI, which was significantly higher than the incidence in the normal blood glucose group [31]. In a prospective study, Uppalakal and Karanayil [32] reported a 40% incidence of metabolic syndrome in STEMI patients, 93% of whom had an elevated FBG level (FBG > 100 mg). Elevated FBG is an independent risk factor for CAD (odds ratio (OR) = 2.238, 95% CI = 1.111–4.508, *P*=0.024); reducing FBG is beneficial for preventing the occurrence and reducing the incidence of CAD [33]. Elevated FBG is associated with the severity of CAD and is correlated with the Gensini score [34]. In a study of 1028 patients who underwent coronary angiography, patients with an elevated FBG level had more severe coronary artery lesions and higher Gensini scores (*P* < 0.001). A meta-analysis [35] also indicated that IFG was an independent risk factor for cardiovascular disease (OR = 1.12–1.37). These results are consistent with those of the present study. However, some authors have reported the opposite findings; a follow-up study of 11338 patients indicated no significant difference in the severity of CAD and the incidence of composite cardiovascular endpoint events in patients with prediabetes compared to the normal population [36]. The FBG and postprandial blood glucose (PBG) were positively correlated with the Gensini score in 1852 patients who underwent coronary angiography (*r* = 0.09, *P* < 0.01; and *r* = 0.20, *P* < 0.01, respectively), whereas regression analysis showed that FBG rather than PBG was independently associated with Gensini score.

This study has several limitations. Firstly, this was a single-center study with a small sample size. A multicenter study with a larger sample size is needed to further evaluate the correlations between RBG, FBG, and Gensini score and to determine the predictive value for short-term and long-term adverse prognoses. Secondly, this study did not explore the correlation between PBG and Gensini score. Thirdly, we did not report the exact HbA1c value. Finally, this study did not clarify whether interventions for hyperglycemia can reverse its adverse effects.

## 5. Conclusions

In conclusion, we should pay more attention to FBG, and timely intervention for hyperglycemia, by using insulin or other glucose-lowering medication, may bring more benefit for patients with myocardial infarction.

## Figures and Tables

**Table 1:** Gensini score rule.

Degree of coronary artery stenosis	Score	Coronary lesion site	Score
≤25%	1	LM	5
26%–50%	2	Proximal LAD or LCX	2.5
51%–75%	4	Middle LAD	1.5
76%–90%	8	Distal LAD	1.0
91%–99%	16	Middle or distal LAD	1.0
100%	32	RCA	1.0
		Subbranch	0.5

LM: left main coronary artery; LAD: left anterior descending; LCX: left circumflex coronary; RCA: right coronary artery.

**Table 2:** Comparison of baseline data, hematological indexes, coronary angiography data, and Gensini score between the hyperglycemia group and nonhyperglycemia group.

Variables	Hyperglycemia group (*n* = 265)	Nonhyperglycemia group (*n* = 693)	*P* value
Sex (man)	193	551	0.026^*∗*^
Age	64.08 ± 12.94	61.64 ± 13.47	0.019^*∗*^
Height (cm)	166.71 ± 7.44	166.78 ± 7.54	0.92
Weight (kg)	68.55 ± 12.18	68.76 ± 11.38	0.844
BMI (kg/m^2^)	24.93 ± 3.87	24.44 ± 3.53	0.132
SBP (mmHg)	124.80 ± 24.80	126.52 ± 20.83	0.315
DBP (mmHg)	76.14 ± 16.29	76.98 ± 14.32	0.472
Hypertension	167	384	0.033^*∗*^
Diabetes	174	88	<0.001^*∗*^
Previous MI	12	10	0.013^*∗*^
Cerebral infarction	37	65	0.040^*∗*^
CKD	30	49	0.032^*∗*^
Smoking	119	380	0.006^*∗*^

*Killip grade*			
I grade	158	502	<0.001^*∗*^
II grade	42	112	0.906
III grade	2	9	0.480
IV grade	63	70	<0.001^*∗*^
TNI P_50_ (P_25_–P_75_) (ng/ml)	17.9 (3.2–25)	14 (3.53–25)	0.286
Myo P_50_ (P_25_–P_75_) (ng/ml)	500 (241–900)	500 (239–900)	0.096
CK-MB P_50_ (P_25_–P_75_) (ng/ml)	80 (26.9–292)	80 (44–303.25)	0.406
BNP P_50_ (P_25_–P_75_) (pg/ml)	216.5 (55.9–862)	134 (43.3–393.25)	0.002^*∗*^
WBC (^*∗*^10^9^/L)	12.46 ± 4.61	11.19 ± 3.68	<0.001^*∗*^
Hb (g/L)	133.21 ± 23.15	135.99 ± 20.71	0.098
PLT (^*∗*^10^9^/L)	207.48 ± 66.48	204.68 ± 60.07	0.564
RBG (mmol/L)	16.34 ± 4.85	7.35 ± 1.62	<0.001^*∗*^
ALT P_50_ (P_25_–P_75_) (IU/L)	53 (34–82)	44 (30–68.75)	0.001^*∗*^
AST P_50_ (P_25_–P_75_) (IU/L)	174.5 (79–323)	157.5 (76.75–262.25)	0.073
Scr P_50_ (P_25_–P_75_) (*μ*mol/L)	81 (71–105.5)	80.5 (69–92)	0.1
eGFR P_50_ (P_25_–P_75_) (ml/min/1.73 m^2^)	77.18 (54.95–96.24)	82.37 (68.89–97.27)	0.022^*∗*^
UA(*μ*mol/L)	364.18 ± 143.79	366.75 ± 115.43	0.079
FBG (mmoL/L)	12.00 ± 5.47	6.45 ± 2.02	<0.001^*∗*^
TG (mmoL/L)	2.12 ± 2.12	1.67 ± 1.15	0.004^*∗*^
TC (mmoL/L)	4.27 ± 1.34	4.48 ± 1.35	0.044^*∗*^
HDL-c (mmoL/L)	1.02 ± 0.25	1.13 ± 0.41	<0.001^*∗*^
LDL-c (mmoL/L)	2.62 ± 0.99	2.80 ± 0.86	0.017^*∗*^
LVEF (%)	0.54 ± 0.12	0.58 ± 0.11	<0.0010^*∗*^
Aspirin	263	681	0.26
Clopidogrel	21	95	0.014^*∗*^
Ticagrelor	244	598	0.014^*∗*^
ACEI/ARB	112	370	0.002^*∗*^
*β*-blocker	214	580	0.28
Statin	265	688	0.166
CCB	16	44	0.338
Furosemide	72	177	0.424
Antisterone	70	179	0.261
IABP	28	35	0.002^*∗*^
CABG	5	8	0.381

*Coronary lesions*			
Single-vessel lesion	44	192	<0.001^*∗*^
Double-vessel lesions	95	191	0.012^*∗*^
Triple-vessel lesions	126	310	0.434

*Number of stents*			
Single stent	167	415	0.374
Two or more stents	44	133	0.356
Left main lesion	37	62	0.023^*∗*^
Gensini score P_50_ (P_25_–P_75_)	71.5 (41–92)	58 (36–87)	0.006^*∗*^

BMI: body mass index; SBP: systolic blood pressure; DBP: diastolic blood pressure; CKD: chronic kidney diseases; TNI: troponin; Myo: myohemoglobin; CK-MB: creatine kinase-MB; BNP: brain natriuretic peptide; WBC: white blood cell; Hb: hemoglobin; PLT: platelet; RBG: random blood glucose; ALT: alanine transaminase; AST: Aspartate transaminase; Scr: serum creatinine; eGFR: estimated glomerular filtration rate; UA: uric acid; FBG: fasting blood glucose; TG: triglyceride; TC: total cholesterol; HDL-c: high-density lipoprotein-cholesterol; LDL-c: low-density lipoprotein-cholesterol; LVEF: left ventricular ejection fraction; CCB: calcium channel blocker; IABP: intra-aortic balloon pump; CABG: coronary artery bypass grafting.

**Table 3:** Comparison of baseline data, hematological parameters, coronary angiography data, and Gensini score between the FBG elevated group and the non-FBG elevated group.

Variables	FBG elevated group (*n* = 589)	Non-FBG elevated group (*n* = 369)	*P* value
Sex (man)	433	303	0.002^*∗*^
Age	62.91 ± 13.01	61.72 ± 13.84	0.185
Height (cm)	166.77 ± 7.64	166.70 ± 7.20	0.087
Weight (kg)	69.02 ± 12.20	68.15 ± 11.73	0.319
BMI (kg/m^2^)	24.77 ± 3.70	24.09 ± 3.71	0.012^*∗*^
SBP (mmHg)	127.08 ± 23.18	125.34 ± 19.14	0.235
DBP (mmHg)	76.89 ± 15.05	76.44 ± 13.84	0.467
Hypertension	349	202	0.169
Diabetes	242	20	<0.001^*∗*^
Previous MI	15	7	0.514
Cerebral infarction	86	16	<0.001^*∗*^
CKD	54	25	0.190
Smoking	283	216	0.002^*∗*^

*Killip grade*			
I grade	367	293	<0.001^*∗*^
II grade	103	51	0.133
III grade	9	2	0.163
IV grade	99	34	<0.001^*∗*^

TNI P_50_ (P_25_–P_75_) (ng/ml)	17 (3.98–25)	11 (2.3–25)	0.002^*∗*^
Myo P_50_ (P_25_–P_75_) (ng/ml)	500 (219.75–900)	500 (206.25–900)	0.016^*∗*^
CK-MB P_50_ (P_25_–P_75_) (ng/ml)	80 (36.45–297)	80 (37.5–246.5)	0.260
BNP P_50_ (P_25_–P_75_) (pg/ml)	173 (49.75–587.5)	112 (41.45–347.5)	0.001^*∗*^
WBC (^*∗*^10^9^/L)	12.29 ± 4.45	10.47 ± 2.94	<0.001^*∗*^
Hb (g/L)	134.5 ± 23.11	135.99 ± 20.71	0.066
PLT (^*∗*^10^9^/L)	209.87 ± 63.42	201.7 ± 58.36	0.051
RBG (mmol/L)	11.25 ± 5.21	7.22 ± 2.35	<0.001^*∗*^
ALT P_50_ (P_25_–P_75_) (IU/L)	48 (33–79)	39 (29–59)	<0.001^*∗*^
AST P_50_ (P_25_–P_75_) (IU/L)	145 (60–219)	171 (89–309)	<0.001^*∗*^
Scr P_50_ (P_25_–P_75_) (*μ*mol/L)	81 (67–98)	80 (72–91)	0.862
eGFR P_50_ (P_25_–P_75_) (ml/min/1.73 m^2^)	80.91 (62.22–97.88)	81.56 (69.16–96.16)	0.704
UA(*μ*mol/L)	376.41 ± 143.79	365.35 ± 122.55	0.152
FBG (mmoL/L)	9.79 ± 4.67	5.22 ± 0.52	<0.001^*∗*^
TG (mmoL/L)	1.83 ± 1.66	1.64 ± 0.98	0.051
TC (mmoL/L)	4.46 ± 1.34	4.42 ± 0.98	0.667
HDL-c (mmoL/L)	1.12 ± 0.44	1.07 ± 0.30	0.046^*∗*^
LDL-c (mmoL/L)	2.77 ± 0.99	2.80 ± 0.82	0.682
LVEF (%)	0.53 ± 0.38	0.68 ± 0.34	<0.001^*∗*^
Aspirin	581	364	0.997
Clopidogrel	61	55	0.028^*∗*^
Ticagrelor	528	314	0.028^*∗*^
ACEI/ARB	277	205	0.01^*∗*^
*β*-blocker	494	300	0.304
Statin	586	367	0.946
CCB	30	30	0.059
Furosemide	174	75	0.002^*∗*^
Antisterone	172	77	0.004^*∗*^
IABP	51	12	0.001^*∗*^
CABG	10	3	0.249

*Coronary lesions*			
Single-vessel lesion	101	135	<0.001^*∗*^
Double-vessel lesions	171	115	0.483
Triple-vessel lesions	284	152	0.034^*∗*^

*Number of stents*			
Single stent	340	242	0.015^*∗*^
Two or more stents	124	53	0.009^*∗*^
Left main lesion	75	24	0.002^*∗*^
Gensini score P_50_ (P_25_–P_75_)	65 (42–92)	52 (32–82)	<0.001^*∗*^

SBP: systolic blood pressure; DBP: diastolic blood pressure; CKD: chronic kidney diseases; TNI: troponin; Myo: myohemoglobin; CK-MB: creatine kinase-MB; BNP: brain natriuretic peptide; WBC: white blood cell; Hb: hemoglobin; PLT: platelet; RBG: random blood glucose; ALT: alanine transaminase; AST: aspartate transaminase; Scr: serum creatinine; eGFR: estimated glomerular filtration rate; UA: uric acid; FBG: fasting blood glucose; TG: triglyceride; TC: total cholesterol; HDL-c: high-density lipoprotein-cholesterol; LDL-c: low-density lipoprotein-cholesterol; LVEF: left ventricular ejection fraction; CCB: calcium channel blocker; IABP: intra-aortic balloon pump; CABG: coronary artery bypass grafting.

**Table 4:** RBG and FBG in different Gensini score groups by the LSD method.

		RBG				FBG			
Group	Group	Mean difference	95% CI		*P* value	Mean difference	95% CI		*P* value
			Lower limit	Upper limit			Lower limit	Upper limit	
1	2	−0.17	−0.13	0.79	0.733	−0.35	−1.12	0.42	0.378
	3	−1.11	−2.05	−0.17	0.020^*∗*^	−0.91	−1.67	−0.14	0.020^*∗*^
	4	−1.75	−2.71	−0.80	<0.001^*∗*^	−1.72	−2.5	−0.94	<0.001^*∗*^
2	3	−0.94	−1.89	0.01	0.051	−0.56	−1.33	0.21	0.152
	4	−1.59	−2.59	−0.62	0.001^*∗*^	−1.37	−2.15	−0.59	0.001^*∗*^
3	4	−0.64	−1.59	0.30	0.183	−0.81	−1.59	−0.04	0.04^*∗*^

RBG: random blood glucose; FBG: fasting blood glucose. ^*∗*^*P* < 0.05.

**Table 5:** Multivariate linear regression analysis for Gensini score as a continuous variable.

Variables	*β*	*P* value
Sex	9.770	0.015^*∗*^
Age	0.125	0.098
BMI	0.05	0.793
Hypertension	1.147	0.701
Diabetes	8.366	0.043^*∗*^
CKD	0.508	0.936
Smoking	2.453	0.467
eGFR	−0.165	0.044^*∗*^
FBG	1.540	0.039^*∗*^
RBG	1.145	0.112
TG	0.409	0.138
TC	2.254	0.252
HDL-c	0.465	0.372
LDL-c	−0.503	0.636

CKD: chronic kidney diseases; eGFR: estimated glomerular filtration rate; RBG: random blood glucose; FBG: fasting blood glucose; TG: triglyceride; TC: total cholesterol; HDL-c: high-density lipoprotein-cholesterol; LDL-c: low-density lipoprotein-cholesterol.

**Table 6:** Multivariate logistic regression analysis for the Gensini score as a hierarchical variable.

Gensini score group	Variables	OR	95% CI	*P* value
*Gensini score* *≤* 37				
37 < Gensini score ≤ 60	Sex	1.20	0.729–1.974	0.473
	Age	1.031	1.013–1.050	0.001^*∗*^
	Diabetes	1.001	0.513–1.812	0.997
	eGFR	1.006	0.000–1.014	0.090
	RBG	0.941	0.878–1.009	0.087
	FBG	1.111	1.006–1.226	0.037^*∗*^
60 < Gensini score ≤ 88	Sex	1.233	0.746–2.039	0.414
	Age	0.99	0.973–1.007	0.260
	Diabetes	0.949	0.573–1.679	0.858
	eGFR	0.99	0.983–0.998	0.014^*∗*^
	RBG	0.959	0.901–1.002	0.199
	FBG	1.067	1.006–1.279	0.001^*∗*^
Gensini score > 88	Sex	0.688	0.400–1.182	0.176
	Age	1.018	0.999–1.037	0.057
	Diabetes	0.615	0.337–1.120	0.112
	eGFR	0.988	0.980–0.996	0.005^*∗*^
	RBG	0.986	0.926–1.050	0.667
	FBG	1.202	1.097–1.316	<0.001^*∗*^

eGFR: estimated glomerular filtration rate; RBG: random blood glucose; FBG: fasting blood glucose. ^*∗*^*P* < 0.05.

## Data Availability

The data that support the findings of this study are available from the corresponding author upon reasonable request.
